# The Regulatory Role of Exogenous Carnitine Applications in Lipid Metabolism, Mitochondrial Respiration, and Germination in Maize Seeds (*Zea mays* L.)

**DOI:** 10.3390/life15040631

**Published:** 2025-04-09

**Authors:** Hulya Turk, Mucip Genisel, Rahmi Dumlupinar

**Affiliations:** 1 Biology Department, Science Faculty, Ataturk University, 25240 Erzurum, Turkey; rahmidumlupinar@atauni.edu.tr; 2 Department of Pharmaceutical Botany, Faculty of Pharmacy, Agri Ibrahim Cecen University, 04100 Agri, Turkey; mgenisel@agri.edu.tr

**Keywords:** carnitine, germination, lipid metabolism, mitochondrial respiration, gene expression, maize

## Abstract

The present study aimed to investigate the effects of exogenous carnitine treatments on maize seed germination by stimulating lipid metabolism and regulating the mitochondrial respiratory pathway. Maize seeds were grown as control, 5, 7.5, and 10 μM carnitine treatment groups in a germination chamber at 25 °C under dark conditions for 5 d. It was determined that carnitine treatments increased the germination rate (GR), germination index (GI), germination potential (GP), vigor index (VI), root and hypocotyl length, fresh weight (FW), and content of total soluble protein but decreased the total carbohydrate content. It was also found that it increased the activities of α-amylase, isocitrate lyase (ICL), and malate synthase (MS) enzymes, which are critical in the germination process, and upregulated the expression of ICL and MS genes. To clarify the potential of carnitine treatments to promote the participation of lipids in respiration in roots and hypocotyls, lipase, carnitine acyltransferases (CATI and CATII), and citrate synthase (CS) enzyme activities were examined, and significant increases in these activities were detected. It was also found that gene levels of respiratory enzymes cytochrome oxidase (COX), pyruvate dehydrogenase (PDH), and Atp synthase, lipase, and CS proteins were upregulated by carnitine treatment. In support of the enzyme and gene change findings, significant changes were determined in fatty acid contents, free carnitine, and long-chain acylcarnitine levels in seeds, roots, and hypocotyls depending on carnitine application. In roots and hypocotyls, carnitine treatments significantly increased glutamine synthase (GS) and glutamate dehydrogenase (NADH-GDH) activities and gene expression levels, which are closely related to the tricarboxylic acid cycle (TCA). It was also noted that all proteins analyzed at the gene expression level were upregulated by carnitine applications in seeds. In addition, significant increases were recorded in antioxidant enzyme ascorbate peroxidase (APX) and superoxide dismutase (SOD) activities and total ascorbate (AsA) and glutathione (GSH) contents in roots and hypocotyls, while decreases were determined in guaiacol peroxidase (GPX) and catalase activities. Significant changes were recorded in all parameters examined, especially with 7.5 µM carnitine application. The findings suggest that carnitine may promote the transport of fatty acids to mitochondrial respiration by accelerating lipid catabolism in five-day-old maize and contribute to seed germination and growth and development processes by activating other metabolic pathways associated with respiration in this process.

## 1. Introduction

Seed germination, which constitutes the beginning of the plant’s life cycle, is a complex physiological process that begins with water uptake by the dry seed and ends with the outgrowth of the rootlet [1,2]. This process has a dynamic structure in which physical and metabolic events work together and are significantly affected by environmental conditions [3,4,5]. Seed germination also has a decisive role in the productivity of agricultural production. In this context, maize is strategically crucial for human nutrition and animal feed and a significant source for producing industrial raw materials, such as starch, glucose, and corn oil [6]. Maize agriculture has an economic value and plays a critical role in ecological and agricultural sustainability due to its contribution to animal protein production [7]. Therefore, studying the germination process of maize and the factors affecting this process is of considerable importance for both agricultural production and industrial applications. In addition, some substances applied exogenously to seeds during the germination stage accelerate the germination process, promote plant development, and increase its economic potential.

Carnitine (4-N-trimethylammonium-3-hydroxybutyric acid), a quaternary ammonium compound broadly found in sources from bacteria to mammals, has various metabolic functions ranging from stress tolerance to detoxification and energy metabolism [8,9]. In mammals, the esterification reaction between the carboxyl groups of activated fatty acids and carnitine is catalyzed by the enzyme CATI [10,11], and the resulting acyl-carnitine molecules can readily pass into mitochondria via carnitine translocase activity [12]. They are then transformed back into acyl-CoA units by the CATII enzyme present in the mitochondrial matrix [10,11,12,13]. This process contributes to the synthesis of ATP, the main cellular energy source, by maintaining the balance of acyl-CoA in the cytoplasm and the matrix through β-oxidation reactions [8]. In addition to this function, carnitine is also associated with various metabolic processes, such as the binding or removal of acyl residues formed due to amino acid metabolism, as well as lipid metabolism [8].

The existence of carnitine in plants was first identified by Fraenkel [14], and subsequent studies have identified the presence and amount of free and acylcarnitine derivatives in tissues and organs of many species, including cereals, legumes, dry and germinating seeds, and leaves [15]. However, the level of carnitine in plant tissues has been found to be about a thousand times lower than in animal tissues [16,17]. As in mammals, the existence of acylcarnitine and CAT activities in plant tissues suggests that carnitine may play an important role in lipid metabolism and peroxisomal and mitochondrial fatty acid β-oxidation processes [18,19,20]. In addition, it has also been reported that carnitine offers an osmoprotective effect under stress conditions due to its structural similarity to glycine and proline [8,21]. In addition, it has been reported that the antioxidant properties observed in animals [22,23,24], fungi [25], and bacteria [26,27] contribute to enhanced tolerance to abiotic stresses in plants. Moreover, carnitine has been reported to have positive effects on various vital mechanisms in plants, such as the maintenance of membrane integrity, the maintenance of photosynthetic activity, and the regulation of the respiration rate [8,21]. When we search the literature, there are limited studies on the effects of carnitine on lipid metabolism in plants. The relationship between carnitine and fatty acid biosynthesis was demonstrated using acetyl-carnitine units as substrates in Arabidopsis chloroplasts [20]. Another study in Arabidopsis revealed that acyl-carnitine derivatives are associated with anabolic lipid metabolism pathways, and it is reported that carnitine plays a critical role in the biosynthesis of membrane and storage lipids. Furthermore, plant carnitine has been implicated in fatty acid mobility and peroxisomal β-oxidation [19]. A recent study focused on elucidating the interactions of carnitine between lipid metabolism and mitochondrial respiration and reported that exogenous application of carnitine increased the transfer of fatty acids into the mitochondrial matrix and promoted mitochondrial respiration in maize leaves under both normal and low-temperature conditions [18].

To our knowledge, this is the first study to investigate the possible stimulatory effects of carnitine on seed germination and early growth stages at physiological, biochemical, and molecular levels by aiming to reveal the interactions of carnitine between lipid metabolism and mitochondrial respiration in maize seeds. The findings provide a significant basis for further studies to understand the possible effects of carnitine.

## 2. Materials and Methods

### 2.1. Seed Germination Status and Carnitine Treatments

In this experiment, maize seeds (*Zea mays* L. cv. Hido) were utilized. Initially, all seeds were subjected to surface sterilization by using 96% ethanol for 1 min and then 5% NaClO for 10 min, and then they were washed with ultrapure water several times. Following the sterilization step, the seeds were split into four different groups: a control group (control), a 5 µM carnitine group (5 C), a 7.5 µM carnitine group (7.5 C), and a 10 µM carnitine group (10 C). The solutions containing these concentrations were prepared by dissolving L-Carnitine hydrochloride (Sigma Aldrich, Saint Louis, MO, USA, ≥97.0% (HPLC)) in high pure water (Lot Number Z0727533112, LC-MS Grade, Supelco, Burlington, VT, USA). Approximately 6 h after the imbibition, seeds were planted into Petri dishes (10 × 10 cm) that had a double layer of filter paper (Whatman No. 1) damped with 10 mL of each group’s own solutions. Eight seeds were planted in a Petri dish. All sown seeds were grown in a germination cabinet in the dark at 25 °C for 5 d. On the fifth day, all seedlings were harvested and stored at −86 °C until the determination of changes in physiological, biochemical, and molecular parameters.

### 2.2. Germination Parameters

All seeds in Petri dishes were checked daily, and their germination percentage was determined by counting the germinated seeds. The numbers of germinated seeds on day 5 were used for the germination rate (GR) and the germination potential (GP). The germination index (GI) was determined according to the method described by Wang et al. [28], GI = Σ (Gi/Ti), where Ti is the day of the germination test and Gi is the number of germinated seeds per day corresponding to Ti. The vigor index (VI) was determined using the formula given as VI = GI × fresh weight (FW) [29]. The fresh weight of all groups was recorded on the fifth day. Germination parameters were measured with five repetitions.

### 2.3. Root and Hypocotyl Lengths

After harvest, the lengths of the roots and the hypocotyls of maize seedlings were determined by using the centimetric scale [30].

### 2.4. Total Soluble Protein and Carbohydrate Contents

The content of total soluble protein was determined in accordance with Smith et al. [31] by using the bicinchoninic acid (BCA) technique. The content of total carbohydrates was determined with respect to Dische [32].

### 2.5. α-Amylase, Isocitrate Lyase (ICL), and Malate Synthase (MS) Activities

Amylase activity was recorded at 620 nm according to the method described by Juliano and Varner [33]. One unit of amylase activity was assigned as the amount of enzyme that caused the changes in the absorbance of 0.1 [34].

ICL activity was measured through the procedure of Dixon and Kornberg [35] at 324 nm. The molar extinction coefficient for (1.7 × 10^4^ cm^−1^ M^−1^) was used for ICL enzyme activity. MS activity was carried out with respect to Hock and Beevers [36] at 412 nm. Seeds were used for these enzymes’ activities.

### 2.6. Lipase Activity

One g of root and hypocotyl tissues were used for the determination of lipase activity. The absorbance changes were measured at 340 nm at room temperature for 5 min according to Zhong and Glatz [37], with minor modification [18]. Different concentrations of nitrophenol solution were used in preparing the standard curve. One unit of lipase activity was stated as the amount of enzyme that caused the release of nitrophenol 1 µmol min^−1^.

### 2.7. Saturated and Unsaturated Fatty Acid Contents

The samples of root, hypocotyl, and seed were dried for 72 h in the oven. Later, 2 mL of hexane was added into the dried 0.1 g tissue sample and sonicated for 5 min. After this period, 2 mL of 300 mM KOH was added into all samples and sonicated again for 5 min. All samples were incubated at room temperature by shaking for one hour. All tissue samples were filtered by using a 0.45 micron filter and analyzed at the GC-FID with FAME mix 37 (Supelco, Burlington, VT, USA) as the reference. This procedure was performed by the Eastern Anatolia High Technology Application and Research Center (DAYTAM).

### 2.8. Citrate Synthase (CS) Activity

Citrate synthase activity was determined according to the producer’s instructions with the Citrate Synthase Assay Kit (Catalog Number CS0720 Sigma-Aldrich). Firstly, mitochondria isolation was performed with this Kit, and then CS activity was measured spectrophotometrically and calculated using the coefficient of Ɛ412 = 13.6 cm^−1^ mM^−1^ [38]. Citrate synthase activity was determined in root and hypocotyl tissues.

### 2.9. Carnitine Acyltransferase (CATI and CATII) Activities

The CATI and CATII activities were measured based on the method of Schwabedissengerbling and Gerhardt [39], with some modifications [18], in root and hypocotyl mitochondria. To determine CATI activity, the absorbance changes of tissue samples were noted at 412 nm spectrophotometrically, and an extinction coefficient of 13.6 cm^2^ µM^−1^ was used. The absorbance values of samples were noted at 232 nm. CATII activity was determined according to the coefficient of Ɛ232 = 5.0 cm^2^ M^−1^. Long-chain oleoyl CoA was used as a substrate instead of acetyl CoA for CATI. Long-chain palmitoyl CoA was used as specified in the method for CATII. Carnitine acyl transferase activities were determined in root and hypocotyl tissues.

### 2.10. Free L-Carnitine and Acylcarnitine Contents

The contents of free L-carnitine and acyl-carnitine were measured by using the technique of Bourdin et al. [40] with minor alterations [18] using an LC-MS/MS device. The HPLC separation was performed on an Agilent Zorbax SB-C8 Solvent Saver Plus column (3.0 × 1500 mm 3.5-Micron- Agilent Technologies, Santa Clara, CA, USA) and an Agilent HILIC column (3.0 × 150 mm 4,6-Micron). The mass spectrometry analysis was performed in Agilent 6460 Triple Quadropol-LC MS/MS (Agilent Technologies, Santa Clara, CA, USA) using the positive MRM mode. Decanoyl-L-Carnitine (C10-Carnitine, ≥97.0% (TLC), CAS No: 3992-45-8), Palmitoyl-L-Carnitine (C16-Carnitine, ≥97.0% (TLC), CAS No: 2364-67-2), Stearoyl-L-Carnitine (C18-Carnitine, ≥97.0% (TLC), CAS No: 25597-09-5), and L-carnitine (≥97.0% (HPLC), CAS No: 6645-46-1) were provided from Sigma-Aldrich (Saint Louis, USA). The amount of free carnitine and acyl carnitine derivatives was determined using the area under each certain peak with an apparatus program. The quantification of the sample concentration was determined using linear regression. Transitions corresponding to standards are given in Table 1. L-carnitine and acyl carnitine derivatives were determined in the roots, hypocotyl, and seeds. This procedure was performed by the Technology Research Development Application and Research Center (TUTAGEM).

### 2.11. RNA Isolation and Real-Time PCR Analysis

The isolation of total RNA of all tissues was conducted in a Qiacube device according to the producer’s analysis protocol by utilizing the RNeasy plant mini kit (Qiagen, Hilden, Germany). The plant samples’ RNA naivety was analyzed through the Qiaexpert. For cDNA synthesis, a Nanoscript 2 RT kit (Primer Design, Hilden, Germany) was utilized. A Thermal Cycler (Qiagen, Rotor-Gene Q, Hilden, Germany) was used for the amplification reactions. The qRT-PCR arrangement contained these settings: (1) 10 min of first denaturation at 95 °C, (2) 40 cycles of denaturation at 95 °C for 10 s, (3) 30 s of annealing at 56 °C, and (4) 30 s extension at 72 °C. In this experiment, the β-actin gene was preferred as a housekeeping gene. Rotor-Gene Q Series Software (Software version no: 2.3.5 (Build 1)) was used to obtain the Ct (Cycle threshold) data. All specific gene primers studied were acquired from the Qiagen Firm. The heat map examination was performed using Qiagen-Geneglobe - Website (http://www.qiagen.com/geneglobe, accessed on 26 August 2024). All primer series of the genes studied are given in Table 2.

### 2.12. Glutamate Dehydrogenase (NADH-GDH) and Glutamine (GS) Activities

Glutamate dehydrogenase (GDH) activity was detected by measuring the oxidation of NADH (aminating GDH activity, NADH-GDH) as defined by the technique of Groat and Vance [41]. GDH enzyme’s one unit was computed in units of μmol of NADH oxidized per minute. Glutamine synthetase (GS) activity was measured utilizing the technique of O’neal and Joy [42]. One unit of GS activity was defined as 1 µmol min^−1^ of glutamyl hydroxamate production.

### 2.13. Antioxidant Enzyme Activities and Non-Enzymatic Compounds

SOD activity was analyzed through the inhibition of the photochemical reduction of nitro blue tetrazolium chloride [43]. GPX activity was determined by measuring the enhancement in color alteration at 470 nm [43]. Catalase activity was assayed at 240 nm depending on the H_2_O_2_ decomposition rate using the extinction coefficient of 40 mM^−1^ cm^−1^ [44]. APX activity was determined using an extinction coefficient of 2.8 mM^−1^ cm^−1^ at 290 nm [45].

The total AsA analysis was performed with slight modifications in accordance with Hodges et al. [46]. The analysis of total GSH was carried out in accordance with Wu et al. [47], and it was revised using the procedure of Hodges et al. [46].

### 2.14. Statistical Analysis

All results are the average of fifteen values obtained from five independent experiments with three repetitions for each sample. Variance analysis (ANOVA) was utilized to compare significant dfferences between the sample means. Duncan’s multiple range method (*p* < 0.05) was used to identify statistical significance. The SPSS 20.0. program was chosen to carry out statistical analysis. All figures and tables are presented with the standard errors. Ct values were entered into an Excel file to form a table for gene analysis. The analysis report of the clustergram was obtained from the QIAGEN Company web portal at GeneGlobe (http://www.qiagen.com/geneglobe, accessed on 26 August 2024). All Ct values obtained were standardized depending on Manual Selection of reference genes.

## 3. Results

### 3.1. Effect of Carnitine on Germination Rate, Germination Potential, Germination Index, Vigor Index, Fresh Weight, and Root and Hypocotyl Length

**Figure 1:**
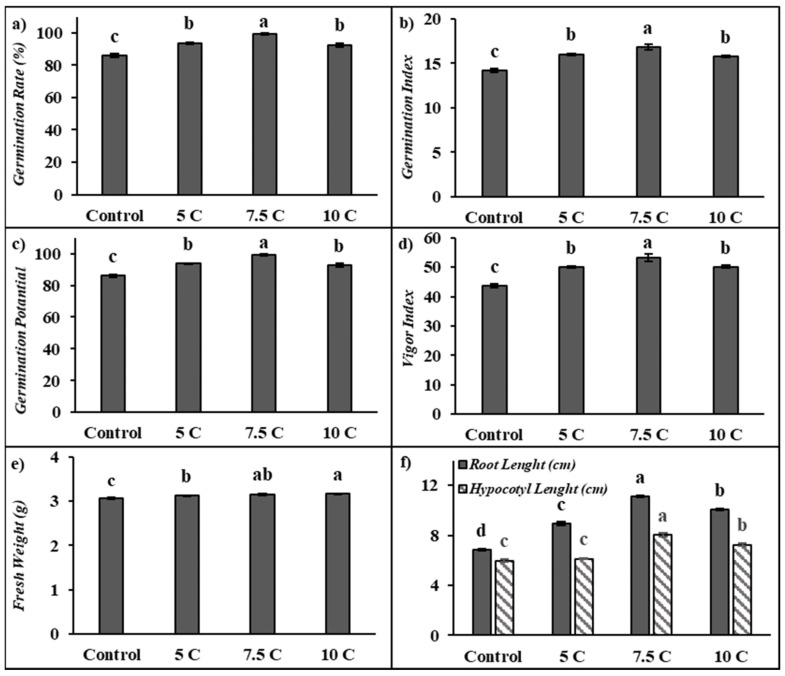
Effects of exogenous carnitine applications on germination rate (GR) (**a**), germination index (GI) (**b**), germination potential (GP) (**c**), vigor index (VI) (**d**), fresh weight (**e**), and root–hypocotyl length (**f**) in the seeds of 5-day-old maize seedlings (5 C: 5 µM carnitine; 7.5 C: 7.5 µM carnitine; 10 C: 10 µM carnitine). Different letters in the figure indicate significant differences within values by Duncan′s test at *p* < 0.05.

As seen from the results summarized in Figure 1, carnitine applications significantly affected the germination rate, germination potential, germination index, vigor index, fresh weight, and root and hypocotyl length of maize seedlings in comparison to the control. The germination rate of seeds under 5, 7.5, and 10 C treatments increased by 8.91%, 15.16%, and 7.56%, respectively (Figure 1a). Figure 1b shows that the germination index increased by 12.66%, 18.39%, and 10.92% separately under 5, 7.5, and 10 C treatments when compared to the control. Similarly, 5, 7.5, and 10 C applications resulted in a remarkable elevation in germination potential by 8.91%, 15.16%, and 7.56%, respectively (Figure 1c). Also, 5, 7.5, and 10 C applications resulted in rises in the content of the vigor index by 14.91%, 22.23%, and 14.95%, respectively, in comparison to the control (Figure 1d). However, there were no significant differences between 5 and 10 C treatments for all of these parameters. The fresh weight of seeds under 5, 7.5, and 10 C treatments increased by 1.98%, 3.18%, and 3.61%, respectively, compared with the control (Figure 1e). There were no significant differences between 7.5 and 10 C treatments for fresh weight. This study showed that carnitine applications exhibited significant increases in root and hypocotyl length in 5-day-old maize seedlings. As indicated in Figure 1f, in comparison to the control, root lengths were markedly increased by 30.93%, 62.62%, and 47.18%; the hypocotyl lengths were increased up to 2.16%, 33.95%, and 21.20% under the 5, 7.5, and 10 C treatments, respectively. While maize seeds treated with all carnitine groups exhibited important increases in the germination tests in comparison to their control, except for hypocotyl lengths in the control and 5 C groups, the highest values were recorded in 7.5 C.

### 3.2. Effect of Carnitine on Total Soluble Protein Content and Total Carbohydrate Content

The contents of total soluble protein and carbohydrates were evaluated in seed tissues compared to the control. As seen in Table 3, the contents of total soluble protein were remarkably increased by 13.58%, 23.82%, and 11.52% under the 5, 7.5, and 10 C applications, respectively. The values of the total soluble protein contents were recorded as 12.79, 14.53, 15.84, and 14.26 mg g^−1^ FW in the control, 5, 7.5, and 10 C applications, respectively. In seed tissues, 5, 7.5, and 10 C applications decreased by 15.09%, 25.67%, and 20.29% in the contents of total carbohydrates, respectively (Table 3). The values of the total carbohydrate contents were determined to be 137.62, 116.85, 102.30, and 109.70 mg g^−1^ FW in the control, 5, 7.5, and 10 C applications, respectively.

### 3.3. Effect of Carnitine on Enzyme Activities of α-Amylase, Isocitrate Lyase (ICL), and Malate Synthase (MS)

The activities of α-amylase, ICL, and MS are presented in Table 3. Carnitine applications exhibited significantly increases in the seed of maize with regard to the control of all of the enzymes studied. Carnitine applications were enhanced by 13.50%, 26.04%, and 12.66% amylase activity, respectively. The values of the α-amylase activity were determined to be 73.23, 83.11, 92.29, and 82.50 U mg protein^−1^ FW in the control, 5, 7.5, and 10 C applications, respectively. As shown in Table 3, ICL activity was increased by 32.63%, 67.40%, and 51.05% under carnitine applications. The values of the ICL activity were determined to be 1.34, 1.78, 2.24, and 2.02 U mg protein^−1^ FW in the control, 5, 7.5, and 10 C applications, respectively. MS activity caused remarkable increases by 31.71%, 70.44%, and 43.45%. The values of the MS activity were determined to be 7.20, 9.49, 12.28, and 10.33 U mg protein^−1^ FW in the control, 5, 7.5, and 10 C applications, respectively (Table 3).

### 3.4. Effect of Carnitine on Lipase Activity

**Figure 2:**
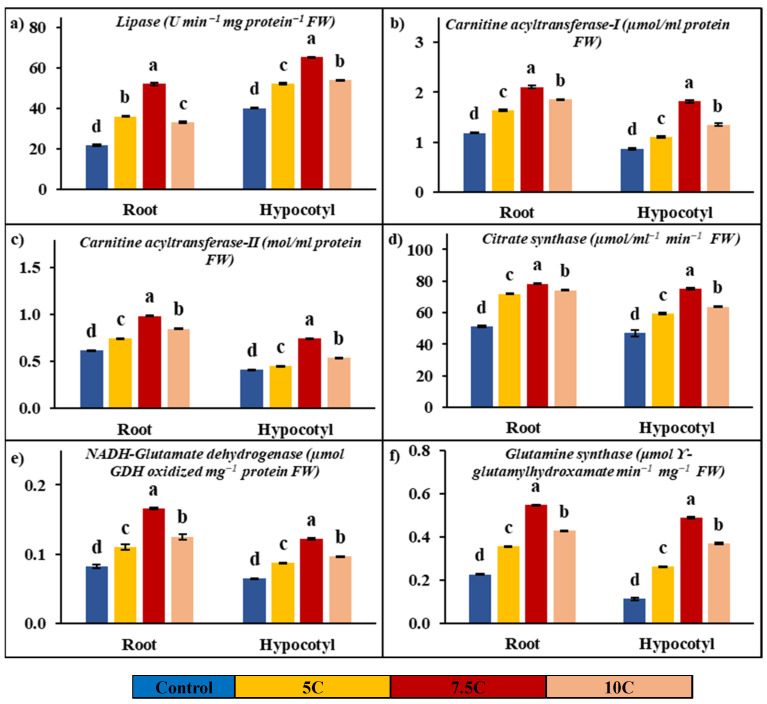
Effects of exogenous carnitine applications on activities of lipase (**a**), carnitine acyltransferase I (CATI) (**b**), carnitine acyltransferase II (CATII) (**c**), citrate synthase (CS) (**d**), glutamate dehydrogenase (NADH-GDH) (**e**), and glutamine synthase (GS) (**f**) in the roots and hypocotyls of 5-day-old maize seedlings (5 C: 5 µM carnitine; 7.5 C: 7.5 µM carnitine; 10 C: 10 µM carnitine). Different letters in the figure indicate significant differences within values by Duncan′s test at *p* < 0.05.

Lipase activity was determined to be affected by carnitine applications in both roots and hypocotyls when compared to the control. As seen in Figure 2a, carnitine treatments led to increases of 65.83%, 139.31%, and 51.94% in roots’ lipase activity, respectively. The increases of 35.91%, 49.64%, and 42.80% in lipase activity were determined in hypocotyls supplied with application of 5, 7.5, and 10 C, respectively. The activities of the root’s lipase were 21.79 U min^−1^mg protein^−1^ FW in the control, 36.13 U min^−1^mg protein^−1^ FW in the 5 C application, 52.14 U min^−1^mg protein^−1^ FW in the 7.5 C application, and 33.10 U min^−1^mg protein^−1^ FW in the 10 C application, respectively. In hypocotyls, the lipase activity was determined to be 40.18, 52.27, 65.23, and 53.88 U min^−1^mg protein^−1^ FW in the control, 5, 7.5, and 10 C applications, respectively.

### 3.5. Effect of Carnitine on Carnitine Acyl-Transferase (CATI and CATII) Activities

Figure 2b,c show the positive effects of carnitine applications on carnitine acyltransferase activities. While 5, 7.5, and 10 C applications increased by 37.42%, 77.47%, and 56.35% in CATI, CATII activity was enhanced by 20.38%, 59.82%, and 37.28% compared to the control in roots, respectively. In hypocotyls, while CATI activity was recorded at 27.27%, 108.72%, and 54.90%, CATII activity was determined to be 9.53%, 81.57%, and 30.86% in 5, 7.5, and 10 C applications in comparison to the control, respectively. In roots, the activities of CATI and CATII were 1.19 μmol/mL protein and 0.62 mol/mL protein in the control, 1.63 μmol/mL protein and 0.74 mol/mL protein in the 5 C application, 2.11 μmol/mL protein and 0.99 mol/mL protein in the 7.5 C application, and 1.85 μmol/mL protein and 0.85 mol/mL protein in the 10 C application, respectively. In hypocotyls, while the values of CATI activity were recorded as 0.87, 1.11, 1.82, and 1.35 µmol/mL protein, CATII activity values were determined to be 0.41, 0.45, 0.75, and 0.54 mol/mL protein in the control, 5, 7.5, and 10 micromolar carnitine applications, respectively.

### 3.6. Effect of Carnitine on Citrate Synthase (CS) Activity

Figure 2d shows significant increases in CS activity with the applications of carnitine in both roots and hypocotyls. In roots, CS activity was increased by 40.53%, 53.03%, and 44.96% in 5, 7.5, and 10 C applications compared to the control, respectively. In hypocotyls, CS activity was enhanced by 26.21%, 59.60%, and 35.40% in 5, 7.5, and 10 C applications with respect to the control, respectively. While the changes were recorded as 51.21, 71.96, 78.36, and 74.23 µmol/mL^−1^ min^−1^ FW in roots, the CS values of hypocotyls were recorded as 47.06, 59.39, 75.10, and 63.72 µmol/mL^−1^ min^−1^ FW in the control, 5, 7.5, and 10 C applications, respectively.

### 3.7. Effect of Carnitine on Glutamate Dehydrogenase (NADH-GDH) and Glutamine Synthase (GS) Activities

Figure 2e,f illustrate the effects of carnitine applications on NADH-GDH and GS activities in both roots and hypocotyls. All results below are presented in comparison to the control. NADH-GDH activities were increased by 33.97%, 102.17%, and 52.60% in roots, and the values of activities of NADH-GDH were recorded as 0.07, 0.09, 0.13, and 0.10 µmol GDH oxidized mg^−1^ protein FW in the control, 5, 7.5, and 10 C applications, respectively. In hypocotyls, NADH-GDH activities were enhanced by 35.04%, 88.35%, and 49.47%, and the values of activities of NADH-GDH were determined to be 0.05, 0.07, 0.10, and 0.08 µmol GDH oxidized mg^−1^ protein FW in the control, 5, 7.5, and 10 C applications, respectively.

In roots, GS activity was raised by 57.06%, 142.87%, and 89.01% in the 5, 7.5, and 10 C applications, respectively. In hypocotyls, GS activity was enhanced by 134.93%, 337.46% and 232.03% in 5, 7.5, and 10 C, respectively. While the changes were recorded as 0.23, 0.35, 0.55, and 0.43 μmol ϒ-glutamyl hydroxamate min^−1^ mg^−1^ FW in roots, the GS values of hypocotyls were recorded as 0.11, 0.26, 0.49, and 0.37 μmol ϒ-glutamyl hydroxamate min^−1^ mg^−1^ FW in the control, 5, 7.5, and 10 C applications, respectively.

### 3.8. Effect of Carnitine on Fatty Acid Contents

In this manuscript, carnitine’s effects on the contents of saturated and unsaturated fatty acids in 5-day-old maize roots, hypocotyls, and seeds were investigated (Table 4). The carnitine treatments promoted the contents of saturated fatty acid (palmitic acid (C16:0), stearic acid (C18:0), and unsaturated fatty acids (alfa-linoleic acid (C18:3n3) and eicosenoic acid (C20:1)) in maize. The increases in palmitic acid, stearic acid, alfa-linoleic acid, and eicosenoic acid contents were recorded with carnitine applications with regard to the control in all parts of the plants. The important changes were recorded for the 7.5 C treatment. In the 7.5 C application, the increased rates of palmitic acid, stearic acid, alfa-linoleic acid, and eicosenoic acid were 3.13%, 45.97%, 29.39%, and 5.55% for seeds and 6.98%, 74.78%, 27.46%, and 16.20% for hypocotyls, respectively. The increased rates of palmitic acid, stearic acid, and alfa-linoleic acid were 7.13%, 65.96%, and 7.86%, and the decreased rate of eicosenoic acid was 34.15% for the roots, respectively.

### 3.9. Effect of Carnitine on Free Carnitine and Acyl-Carnitine Contents

**Figure 3:**
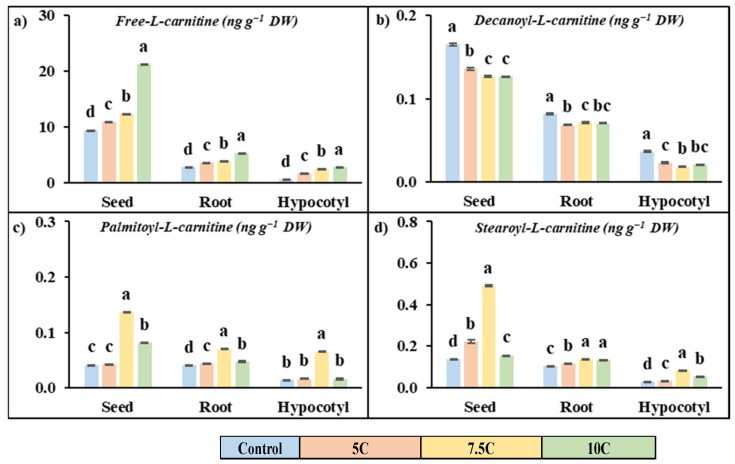
Effects of exogenous carnitine applications on the contents of free-L-carnitine (**a**), decanoyl-L-carnitine (**b**), palmitoyl-L-carnitine (**c**), and stearoyl-L-carnitine (**d**) in seed, root, and hypocotyl tissues of 5-day-old maize seedlings (5 C: 5 µM carnitine; 7.5 C: 7.5 µM carnitine; 10 C: 10 µM carnitine). Different letters in the figure indicate significant differences within values by Duncan′s test at *p* < 0.05.

Carnitine applications had a promoting effect on the free carnitine content in seeds, roots, and hypocotyls of maize with respect to the control (Figure 3a). In 5, 7.5, and 10 C treatments, the increased rates of free carnitine were 32.72%, 44.48%, and 93.93% for seeds, 16.31, 30.85, and 125.10% for roots, and 202.06%, 357.77%, and 401.77% for hypocotyls, respectively.

Carnitine treatment generally increased the content of long-chain acylcarnitine (palmitoyl-L-carnitine and stearoyl-L-carnitine) but decreased the medium-chain acylcarnitine content (decanoyl-L-carnitine) compared to the control in all parts of the plant (Figure 3b–d). The high values were recorded for the 7.5 C treatment. For the 7.5 C application, the increased rates of palmitoyl-L-carnitine were 2.65%, 230.08%, and 96.06% for seeds, 9.11%, 73.17%, and 17.71% for roots, and 18.54%, 367.67%, and 16.30% for hypocotyls, respectively. The increased rates of stearoyl-L-carnitine were 61.11%, 256.27%, and 12.80% for seeds, 12.98%, 32.50%, and 28.48% for roots, and 19.30%, 194.08%, and 91.65% for hypocotyls, respectively. Carnitine treatment did not exhibit a stimulating effect on decanoyl-l-carnitine content. For the 7.5 C treatment, the declining rates of decanoyl-L-carnitine contents were 17.77%, 23.32%, and 23.50% for seeds, 26.24%, 12.58%, and 13.75% for roots, and 37.39%, 50.57%, and 43.72% for hypocotyls, respectively.

### 3.10. Effect of Carnitine on Superoxide Dismutase (SOD), Guaiacol-Peroxidase (GPX), Catalase and Ascorbate Peroxidase (APX) Activities, and Total Ascorbate (AsA) and Glutathione (GSH) Concentration

**Figure 4:**
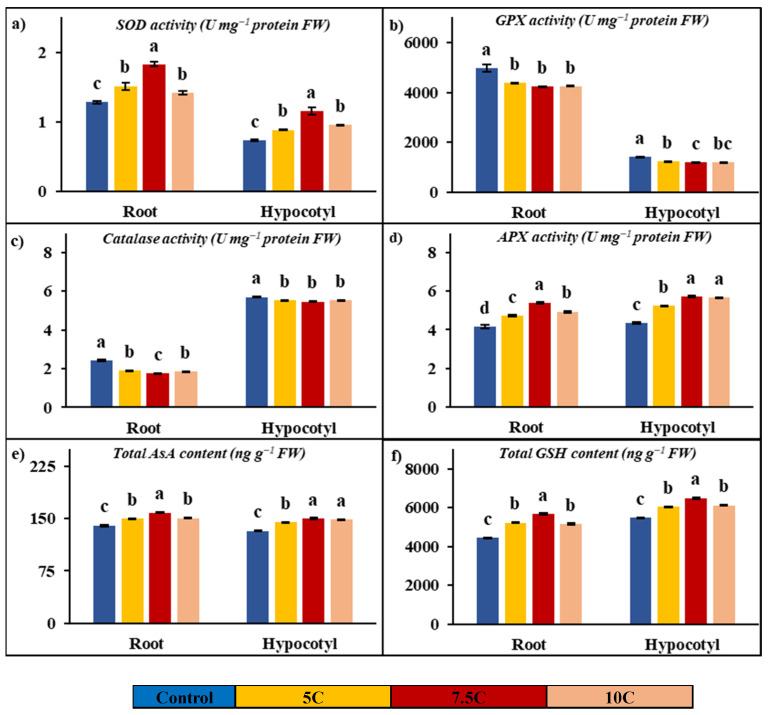
Effects of exogenous carnitine applications on activities of superoxide dismutase (SOD) (**a**), guaiacol peroxidase (GPX) (**b**), catalase (**c**), ascorbate peroxidase (APX) (**d**), total ascorbate (AsA) (**e**), and total glutathione (GSH) (**f**) in the roots and hypocotyls of 5-day-old maize seedlings (5 C: 5 µM carnitine; 7.5 C: 7.5 µM carnitine; 10 C: 10 µM carnitine). Different letters in the figure indicate significant differences within values by Duncan′s test at *p* < 0.05.

Antioxidant enzyme analyses were conducted on SOD, GPX, catalase, and APX enzymes in plants subjected to carnitine application. As indicated in Figure 4a–d, the activities of SOD and APX were enhanced after carnitine treatment, while catalase and GPX activities were decreased in contrast to the control. For the 5, 7.5, and 10 C treatments, the increased rates of SOD activities were 18.33%, 42.41%, and 10.38% for roots and 19.84%, 56.86%, and 29.78% for hypocotyls, respectively. While the changes were recorded as 1.28, 1.52, 1.83, and 1.41 Umg^−1^ protein FW in roots, the SOD values of hypocotyls were recorded as 0.74, 0.88, 1.16, and 0.96 U mg^−1^ protein FW in the control, 5, 7.5, and 10 C applications, respectively. For the 5, 7.5, and 10 C treatments, the increased rates of APX activities were 14.10%, 29.66%, and 18.88% for roots and 20.36%, 31.27%, and 30.30% for hypocotyl, respectively. The values of APX were determined to be 4.15, 4.73, 5.38, and 4.93 U mg^−1^ protein FW in roots and 4.35, 5.23, 5.70, and 5.66 U mg^−1^ protein FW in hypocotyls for the control, 5, 7.5, and 10 C applications, respectively. GPX activities were decreased by 12.14%, 15.08%, and 14.62% in roots, and the values of activities of GPX were recorded as 4971.38, 4368.07, 4221.51, and 4244.68 U mg^−1^ protein FW in the control, 5, 7.5, and 10 C applications, respectively. In hypocotyls, GPX activities were decreased by 13.33%, 16.20%, and 14.98%, and the values of activities of GPX were recorded as 1420.41, 1231.09, 1190.37, and 1207.64 U mg^−1^ protein FW in the control, 5, 7.5, and 10 C applications, respectively. Catalase activities were decreased by 21.61%, 28.12%, and 23.89% in roots, and the values of activities of catalase were determined to be 2.43, 1.90, 1.74, and 1.85 U mg^−1^ protein FW in the control, 5, 7.5, and 10 C applications, respectively. In hypocotyls, catalase activities were decreased by 3.10%, 4.15%, and 3.10%, and the values of activities of catalase were recorded as 5.70, 5.52, 5.46, and 5.52 U mg^−1^ protein FW in the control, 5, 7.5, and 10 C applications, respectively.

Figure 4e,f clearly show the increases in total AsA and GSH contents with carnitine applications in comparison to the control. Carnitine treatment resulted in further increases by 7.17%, 13.01%, and 7.30% in roots; the amounts of AsA concentration were recorded as 140.34, 150.40, 158.60, and 150.59 ng g^−1^ FW in the control, 5, 7.5, and 10 C applications, respectively. Total AsA concentrations were increased by 9.21%, 13.27%, and 12.31% in hypocotyls; the values of this compound’s contents were recorded as 132.41, 144.61, 149.98, and 148.71 ng g^−1^ FW in the control, 5, 7.5, and 10 C applications, respectively. Total GSH concentrations were increased by 17.91%, 28.02%, and 16.39% in roots; the values of this compound’s contents were found to be 4436.00, 5230.58, 5678.83, and 5162.97 ng g^−1^ FW in the control, 5, 7.5, and 10 C applications, respectively. Carnitine treatment provided increases by 10.21%, 18.49%, and 11.80% in hypocotyls; the amounts of GSH concentration were determined to be 5477.80, 6037.10, 6490.65, and 6124.00 ng g^−1^ FW in the control, 5, 7.5, and 10 C applications, respectively.

### 3.11. Effect of Carnitine on Expression of Isocitrate Lyase, Malate Synthase, Lipase, Citrate Synthase, Cytochrome Oxidase, Pyruvate Dehydrogenase, ATP Synthase, Glutamate Dehydrogenase, and Glutamine Synthase

**Figure 5:**
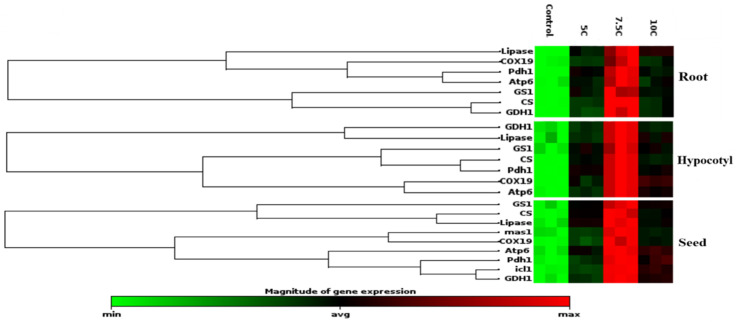
Effects of exogenous carnitine on the gene expression of lipase, citrate synthase, cytochrome oxidase, pyruvate dehydrogenase, ATP synthase, glutamate dehydrogenase, and glutamine synthase in the seeds, roots, and hypocotyls of 5-day-old maize seedlings. Additionally, exogenous carnitine’s effect on the gene expression levels of isocitrate lyase and malate synthase gene expression levels in seeds is shown. The clustergram performs non-supervised hierarchical clustering of the entire dataset to display a heat map with dendrograms indicating co-regulated genes across groups.

The gene expression levels of these enzymes were also studied to clarify the effects of carnitine supplementation on maize seeds, roots, and hypocotyls (Figure 5). In comparison to the control, isocitrate lyase and malate synthase gene levels were increased under the carnitine supplementations, similarly to enzyme activities, as well (Table 3). Citrate synthase, lipase, cytochrome oxidase, pyruvate dehydrogenase, ATP synthase, glutamate dehydrogenase, and glutamine synthase genes were analyzed in seed tissue apart from isocitrate lyase and malate synthase genes, too. These gene levels were upregulated by carnitine treatments with respect to the control (Figure 5). In parallel to the changes in their enzyme activities, citrate synthase, lipase, glutamate dehydrogenase, and glutamine synthase protein transcription levels were increased by carnitine applications in both roots and hypocotyls (Figure 2a–f). Furthermore, cytochrome oxidase, pyruvate dehydrogenase, and ATP synthase gene levels were highly expressed by carnitine applications in comparison to the control in both roots and hypocotyls (Figure 5).

## 4. Discussion

Seed germination, the first phase of the plant life cycle, is a critical physiological process that involves a complex series of physical and biochemical events. This physiological process can be regulated by many plant growth regulators, phytohormones, or bioregulators, including amino acids, such as proline, glutamate, tryptophan, and methionine [29,48]. Bioregulators induce antioxidant metabolism, regulate development, and can act by acting as precursors of plant hormones [49,50]. For this purpose, carnitine, an essential quaternary ammonium compound synthesized from the amino acids lysine and methionine and responsible for energy metabolism in all living organisms, was used as a bioregulator in the present study.

In this study, it was found that exogenous carnitine treatments accelerated the seed germination rate and significantly increased the germination index, vigor index, and germination potential compared to the control. There is a limited number of studies in the literature showing that carnitine increases the germination rate. Birol [51] reported that 1 mM carnitine increased the germination rate of barley seeds under different saline concentrations (0, 0.25, 0.30, and 0.35 M). In another study, 5 μM carnitine application was also found to increase the germination rate in Arabidopsis plants exposed to 150 mM NaCl [8]. However, to our knowledge, there are no studies in the literature showing the effect of carnitine on GI, GP, and VI. Carnitine treatments significantly enhanced the root and hypocotyl length of five-day-old maize seedlings with respect to the control group (Figure 1e). In particular, 7.5 C treatment was found to provide the most effective curative effect on root and hypocotyl length and germination tests. In a study of Arabidopsis, it was reported that carnitine alone or in a mixture with salt provided a notable increase in root length compared to the control group [52]. Seeds need to absorb a sufficient amount of water to start the germination process. Fresh weight is considered an important physiological indicator that directly reflects water absorption and biomass accumulation of seeds. In this study, it was found that carnitine supplementation significantly raised the fresh weight of seeds compared to the control group (Figure 1e). Dos Santos et al. [48] reported that 100 μM carnitine treatment increased the fresh weight in both roots and stems compared to the control in their study with arugula plants. The seed germination process involves a sequential series of complex chemical and physical events involving the activation of enzymatic systems, the promotion of membrane repair processes, and the breakdown of stored reserve substances [29]. During this process, there is a marked increase in the number of biomolecules, such as soluble proteins, to meet the energy and nutrient needs of the seed. In particular, protein content is considered an important indicator of the vegetative growth and development rate. In the current study, it was determined that carnitine treatments increased the total soluble protein content by affecting metabolic processes during the germination process of maize seeds but caused a significant decrease in total carbohydrate content (Table 3). This suggests that carnitine plays a regulatory role in energy metabolism during germination. In particular, the increase in the activity of α-amylase (Table 3), a critical enzyme for germination, suggests that carnitine treatments promote energy production by accelerating the breakdown of carbohydrates into soluble sugars [53,54]. The highest effect was observed at 7.5 C supplementation, supporting the hypothesis that carnitine plays a key role in metabolizing stored carbohydrates and meeting energy requirements. The glyoxylate cycle plays a crucial role in maize seeds, which are rich in lipid content. ICL and MS in the glyoxysome have a significant impact on storage lipid mobilization during seed germination [55,56]. They are known as important molecular markers indicating the germination status of lipid-rich seeds. These enzymes actively participate in the metabolic reactions of the glyoxylate cycle, a metabolic pathway in which two acetyl-coenzyme-A molecules are converted into succinate. The resulting metabolic intermediates are then transported to the mitochondria and converted into saccharides. These saccharides serve as the primary source of nutrients required for growth until the onset of photosynthetic activity in seedlings. This metabolic transition occurs through a series of reactions involving both the Krebs cycle and the reverse reactions of glycolysis. ICL catalyzes the conversion of isocitrate to succinate and glyoxylate molecules, while MS catalyzes the conversion of glyoxylate to malate. In this study, the 7.5 C treatment in particular increased ICL and MS activities in maize seeds compared to the control group (Table 3). To our knowledge, no research in the literature describes the effects of carnitine on α-amylase, ICL, and MS activity. In addition to ICL and MS enzyme activities, the diagram presented as a heat map clearly shows that carnitine treatments significantly upregulated the gene levels of these proteins (Figure 5). The findings regarding enzyme activities and gene expression levels are consistent with the changes observed in total carbohydrate content, total soluble protein content, fresh weight, hypocotyl and root length, and germination tests.

In the present study, lipase activity was analyzed at both enzyme and gene expression levels to evaluate carnitine’s effects on lipid catabolism and fatty acid transport. Lipase is known as a key enzyme that hydrolyzes triglycerides into fatty acids. It was found that carnitine treatments significantly increased lipase activity in roots and hypocotyls compared to control groups (Figure 2a). In addition, the gene expression levels of lipase were examined, and a significant positive correlation with enzyme activity was revealed (Figure 5). In addition to roots and hypocotyls, gene expression levels of lipase were also analyzed in seeds and were reported to be increased compared to the control group (Figure 5). These findings suggest that carnitine treatments have a stimulatory effect on lipase activity and transcription levels of this protein and that this effect promotes the hydrolysis of lipids to fatty acids. In our prior study, it was reported that carnitine alone and in combination with chilling conditions enhanced both enzyme activity and transcript levels of lipase in maize leaves [18]. In this study, in addition to lipase activity, saturated and unsaturated long-chain fatty acid contents were also determined in the roots, hypocotyls, and seeds of maize seedlings. While saturated fatty acids are primarily associated with energy storage and maintaining membrane structure, contributing to stability, unsaturated fatty acids increase membrane fluidity and act in important signaling roles. As shown in Table 4, carnitine treatments increased the content of saturated long-chain fatty acids palmitic and stearic acid as well as unsaturated long-chain fatty acid α-linoleic acid in root, hypocotyls, and seeds compared to the control. While an increase in the content of eicosenoic acid, an unsaturated long-chain fatty acid, was observed in seeds and hypocotyls, a decrease in this content was determined in the roots. All values obtained were positively correlated with lipase activity. This could be an adaptation to maintain the fluidity of membranes by regulating selective permeability and/or a way to turn to new substrates for respiration. Carnitine also has many other important roles, from participating in the structure of cell and organelle membranes to being precursors of important signaling compounds. The fatty acids formed are first catalyzed by acyl-CoA synthase and transformed into acyl-CoA units. While short- and medium-chain acyl-CoA units can be easily transported into the mitochondrial matrix, long-chain acyl CoAs require the carrier molecule carnitine to cross the inner mitochondrial membrane, and these reactions are catalyzed by carnitine acyltransferase (CAT) enzymes [40]. There are a limited number of studies on the existence and activities of CATI and CATII in plants, and these studies are insufficient in terms of scope and content. A previous study reported that CAT activity increased when long-chain fatty acid content increased in bean hypocotyl mitochondria [57]. Again, our previous study found that carnitine alone, cold, and in combined applications increased CATI and CATII in maize seedlings [18]. In this study, it was found that carnitine treatments significantly increased CATI and CATII activities in the root and hypocotyl of maize seedlings and that CATI activity was higher than CATII in percentage compared to the control groups (Figure 2b,c). This finding suggests that carnitine may stimulate the transport of fatty acids into the mitochondrial matrix by increasing CAT activities in both the root and hypocotyl of maize. Free carnitine and acylcarnitine (decanoyl-L-carnitine, palmitoyl-L-carnitine, and stearoyl-L-carnitine) contents were also evaluated to support these findings. Carnitine treatments increased free carnitine content in the roots, hypocotyls, and seeds of maize. While a decrease in medium-chain acyl-carnitine content was observed, statistically significant increases in long-chain acyl-carnitine content were recorded, as shown in Figure 3. These findings are consistent with our previous data that free carnitine and long-chain acyl-carnitine content increased in maize leaves [18]. In another previous study, an increase in total carnitine content was recorded in Arabidopsis plants with 5 µM carnitine treatment compared to the control [8]. The increased free carnitine content could be attributed to the possible accumulation of carnitine in plant cells through absorption from roots and/or increased carnitine biosynthesis. The increase in long-chain acyl-carnitines by carnitine treatments strongly suggests a role for this molecule in transport. Long-chain fatty-acyl-CoA is involved in energy metabolism, while short- and medium-chain fatty-acyl-CoAs are associated with cellular signaling processes [58]. Based on the positive correlation recorded in lipase, CAT activities, and free and acyl-carnitine contents, it can be concluded that there is a powerful relationship between carnitine content and fatty acid traffic. This transfer process may be related to lipid-linked pathways and/or an increased requirement for a new substrate due to higher energy demand.

In the present study, CS activity and transcription levels of CS and COX proteins were also studied to explain the effect of carnitine on lipid catabolism and mitochondrial respiration. CS, known as the first enzyme of the Krebs cycle, was significantly increased by carnitine treatments in roots and hypocotyls compared to the control groups (Figure 2d) Transcript levels of genes encoding CS and COX, the membrane-bound terminal enzymes in the electron transfer chain (ETC), are indicated in Figure 5. It is highly likely that carnitine-induced increased transcription of CS and COX proteins contributed to the enhancement of germination and plant growth by generating the extra energy needed by carnitine to stimulate TCA and ETC rates. These findings correlated with carnitine-induced increases in lipase and CAT I-II activities, suggesting that carnitine can coordinate between fatty acid transport and mitochondrial respiration. Similarly, in our previous study, carnitine administration increased CS and COX gene levels [18]. In addition, pyruvate dehydrogenase and ATP synthase protein transcription levels were also examined to understand the effects of carnitine on mitochondrial respiration. Pyruvate dehydrogenase is the first constituent enzyme of the pyruvate dehydrogenase multienzyme complex, and it catalyzes the conversion of pyruvate to acetyl-CoA, linking glycolysis with the TCA cycle. In parallel with the increase in PDH gene expression, carnitine had the same effect on ATP synthase gene expression levels (Figure 5). ATP synthase is an important enzyme in the ETC chain that provides energy for cells by generating ATP during cellular respiration [59,60]. These findings suggest that carnitine regulates mitochondrial respiration, leading to increased levels of ATP, which is essential for seed germination and plant growth. Hierarchical cluster analysis based on a stack map clearly revealed the co-regulatory effect of carnitine on protein transcription levels of mitochondrial respiration-related enzymes (Figure 5). In particular, the 7.5 C treatment was the concentration at which the most significant changes were observed.

In this study, changes in the activity of the GDH pathway, which is closely related to the TCA cycle in terms of both the carbon skeleton and ATP requirement of the GS pathway, were examined in both enzyme activities and gene levels in seeds’ gene expression and roots and hypocotyls. NADH-GDH activity was measured in the present study to determine its activity in nitrogen assimilation. The NADH-GDH enzyme catalyzes the formation of glutamate from α-ketoglutarate, while GS is the enzyme that converts glutamate to glutamine [61]. It can be concluded that carnitine-induced increases (Figure 2e,f) in nitrogen assimilation at both activity and gene levels contribute to the promotion of growth and development by increasing the levels of amino acids and various nitrogen-containing compounds. In addition, in the present study, the activities of SOD, GPX, APX, and catalase, which are important enzymes of the glutathione-ascorbate cycle, as well as total AsA and GSH contents, which are non-enzymatic compounds involved in the cycle, were examined in roots and hypocotyls, and significant increases were observed for all parameters, except for GPX and catalase activity (Figure 4). These increases revealed that carnitine, showed its antioxidant effect and/or its impact on stimulating antioxidant mechanisms. There is information in the literature about its positive effect on the antioxidant defense system [62,63].

When the findings are evaluated as a whole, the effects of exogenous carnitine applications on maize seeds were as follows. There were increases in germination test parameters, such as the germination rate, germination potential, germination index, vigor index, root and hypocotyl length, and fresh weight content, as well as an increase in the content of total soluble protein but a decrease in the total carbohydrate content. There was an increase in α-amylase, ICL, and MS enzyme activities and protein transcription levels of ICL and MS enzymes in the seed, as well as increases in CATI and CATII activities in roots and hypocotyls. There were also increases in lipase, CS, NADH-GDH, and GS enzyme activities and gene expression levels of these proteins in all tissues. There were significant changes in internal free and acyl-carnitine and saturated–unsaturated fatty acid contents in all tissues, increases in ATP synthase, PDH, and COX protein expressions in all tissues, increases in antioxidant enzyme activities, SOD, APX, total AsA, and GSH contents, and decreases in GPX and catalase activities in roots and hypocotyls.

As a result, it was observed that the 7.5 C treatment provided the most significant results in all parameters examined. Carnitine may provide the necessary nutrients and energy for germination by accelerating the glyoxylate cycle by stimulating the conversion of lipids to sugars in the seed. It may have positive effects on germination and growth parameters through this mechanism. In addition, the bio stimulant role of carnitine applied at micromolar concentrations may stimulate mitochondrial respiration by accelerating lipid catabolism and contribute to the growth and development of plants through the energy obtained. The antioxidant property of carnitine may protect seeds against damage that may occur during the germination stage, improving and sustaining their overall health. These findings provide a strong indication of the positive effects of carnitine on seed development and growth and may provide important opportunities for potential applications in plant biotechnology.

## Figures and Tables

**Table 1:** The studied standards for acylcarnitine and carnitine analysis and transitions corresponding to the standards.

Transitions Corresponding to Standards	Standards
316.1 > 85.1	Decanoyl-L-Carnitine
402.2 > 85.1	Palmitoyl-L-Carnitine
428.2 > 85.1	Stearoyl-L-Carnitine
162 > 103	L-carnitine

**Table 2:** Primer sequences of the genes studied for RT-PCR analysis.

Name	Target Gene	Primer Sequences
Isocitrate lyase	*icl1* forward	5′-GAGATGGCCAAGAAGCTGTG-3′
	*icl1* reverse	5′-GTAGATGGTGTCCAGGTGCT-3′
Malate synthase	*mas1* forward	5′-TCGACTTCGGCCTCTACTTC-3′
	*mas1* reverse	5′-ATCCTCGCTTCTCTGGAGTG-3′
Citrate synthase	*CS* forward	5′-TGCTCACAGTGGAGTTTTGC-3′
	*CS* reverse	5′-AACACTCTTCGGCCTCTCAA-3′
Cytochrome oxidase	*COX19* forward	5′-CATGAGTGCGACTTGGAGAA-3′
	*COX19* reverse	5′-TCAGGAGATGTACCCGCTTC-3′
Lipase	*Lipase* forward	5′-TTGCTCCGGGAGAGAACTTA-3′
	*Lipase* reverse	5′-CAAGCAGTTCTCGACATCCA-3′
Pyruvate dehydrogenase	*Pdh1* forward	5′-CTCAACATTTCGGCCCTCTG-3’
	*Pdh1* reverse	5′-CATAGTCGCCACGCTTGTAG-3′
ATP synthase	*ATP6* forward	5′-CACTTAACGAGCACCACCAG-3′
	*ATP6* reverse	5′-GGATCCTGCAGACTCTCTCC-3′
Glutamate dehydrogenase	*GDH1 forward*	5′-AGGAAGCTCGTCAACTCCAA-3′
	*GDH1* reverse	5′-AGAGCAGCCTCGTTGAGGTA-3′
Glutamine synthase	*GS1* forward	5′-GCAATCCTGAACCTTTCTCTG-3′
	*GS1* reverse	5′-GATGCTCGCAGTCTCATGTT-3′
β-actin	*actb1* forward	5′-GTGACAATGGCACTGGAATG-3′
	*actb1* reverse	5′-CCATGCTCAATCGGGTACTT-3′

**Table 3:** Effects of exogenous carnitine applications on soluble total protein content, total carbohydrate content, α-amylase, isocitrate lyase, and malate synthase activities in seed tissues in 5-day-old maize seeds.

	Soluble	Total	α-Amylase	Isocitrate Lyase	Malate Synthase
	Protein Content (mg g^−1^ FW)	Carbohydrate Content (mg g^−1^ FW)	(U mg Protein^−1^ FW)	(U mg Protein^−1^ FW)	(U mg Protein^−1^ FW)
Control	12.790 ± 0.186 c	137.623 ± 0.258 a	73.227 ± 0.148 c	1.340 ± 0.043 d	7.203 ± 0.101 d
5 C	14.527 ± 0.161 b	116.850 ± 0.093 b	83.107 ± 0.173 b	1.777 ± 0.028 c	9.487 ± 0.097 c
7.5 C	15.837 ± 0.136 a	102.300 ± 0.130 d	92.293 ± 0.131 a	2.243 ± 0.036 a	12.277 ± 0.056 a
10 C	14.263 ± 0.194 b	109.696 ± 0.123 c	82.497 ± 0.393 b	2.024 ± 0.018 b	10.333 ± 0.083 b

Different letters in the same group indicate statistically significant differences (*p* < 0.05).

**Table 4:** Effects of exogenous carnitine applications on saturated and unsaturated fatty acids in seed, root, and hypocotyl tissues in 5-day-old maize seeds. The values in each sample were expressed as %.

		Palmitic Acid	Stearic Acid	alfa-Linolenic Acid	Eicosenoic Acid
Seed	Control	12.371 ± 0.013 c	2.455 ± 0.214 c	1.092 ± 0.026 c	0.317 ± 0.002 c
	5 C	12.577 ± 0.069 b	2.997 ± 0.116 b	1.250 ± 0.023 b	0.332 ± 0.001 ab
	7.5 C	12.790 ± 0.021 a	3.584 ± 0.044 a	1.413 ± 0.049 a	0.335 ± 0.001 a
	10 C	12.606 ± 0.047 b	3.382 ± 0.116 ab	1.303 ± 0.025 b	0.328 ± 0.001 b
Root	Control	24.545 ± 0.156 c	2.349 ± 0.150 b	3.087 ± 0.006 d	0.289 ± 0.002 a
	5 C	25.457 ± 0.087 b	3.734 ± 0.002 a	3.173 ± 0.018 c	0.277 ± 0.002 b
	7.5 C	26.295 ± 0.115 a	3.898 ± 0.541 a	3.330 ± 0.020 a	0.190 ± 0.002 c
	10 C	25.578 ± 0.248 b	3.504 ± 0.032 a	3.265 ± 0.012 b	0.203 ± 0.0002 d
Hypocotyl	Control	24.333 ± 0.169 c	2.473 ± 0.191 c	11.732 ± 0.229 c	0.287 ± 0.001 c
	5 C	25.156 ± 0.305 b	3.312 ± 0.140 b	13.672 ± 0.120 b	0.288 ± 0.0004 b
	7.5 C	26.0317 ± 0.169 a	4.322 ± 0.065 a	14.954 ± 0.525 a	0.333 ± 0.0004 a
	10 C	25.562 ± 0.102 ab	3.623 ± 0.272 b	13.970 ± 0.096 b	0.294 ± 0.001 b

Different letters in the same group indicate statistically significant differences (*p* < 0.05).

## Data Availability

The data presented in this study are available upon request from the corresponding author.

## Abbreviations

The following abbreviations are used in this manuscript:

GR	Germination rate
GI	Germination index
GP	Germination potential
VI	Vigor index
FW	Fresh weight
ICL	Isocitrate lyase
MS	Malate synthase
CATI	Carnitine acyltransferase I
CATII	Carnitine acyltransferase II
CS	Citrate synthase
COX	Cytochrome oxidase
PDH	Pyruvate dehydrogenase
NADH-GDH	NADH-Dependent Glutamate Dehydrogenase
GS	Glutamine synthase
SOD	Superoxide dismutase
GPX	Guaiacol peroxidase
APX	Ascorbate peroxidase
AsA	Ascorbate
GSH	Glutathione
TCA	Tricarboxylic acid cycle
ETC	Electron transfer chain
Ct	Threshold Cycle
ATP	Adenosine Triphosphate
5 C	5 µM carnitine application
7.5 C	7.5 µM carnitine application
10 C	10 µM carnitine application
